# Phase 3 Randomized Study Investigating the Safety, Tolerability, and Immunogenicity of a Combination Modified Messenger RNA Vaccine Against Influenza and COVID-19 in Healthy Adults

**DOI:** 10.3390/vaccines14080677

**Published:** 2026-08-05

**Authors:** Yanely Pineiro Puebla, Helen Nicholls, David Fitz-Patrick, Lucy E. Horton, Matthew W. Gawel, Reynold Duarte Martinez, Xingbin Wang, Georgina Keep, Xia Xu, Emily Gomme, Ingrid Scully, Pirada Suphaphiphat Allen, Kena A. Swanson, Nadine Salisch, Federico J. Mensa, Ruben Rizzi, Özlem Türeci, Uğur Şahin, Annaliesa S. Anderson, Alejandra Gurtman, Kelly A. Lindert

**Affiliations:** 1 Palm Springs Community Health Center, Miami Lakes, FL 33014, USA; 2 Pfizer Vaccines, Pfizer Ltd., Marlow SL6 6RJ, UK; 3 East-West Medical Research Institute, Honolulu, HI 96814, USA; 4 Pfizer Vaccines, Pfizer Inc., La Jolla, CA 92130, USA; 5 Angels Clinical Research Institute, Inc., Miami, FL 33016, USA; 6 Pfizer Vaccines, Pfizer Inc., Collegeville, PA 19426, USA; 7 Pfizer Vaccines, Pfizer Inc., Pearl River, NY 10965, USA; 8 BioNTech SE, 55131 Mainz, Germany; 9 BioNTech US Inc., Cambridge, MA 02139, USA; 10 Pfizer Vaccines, Pfizer Inc., Cambridge, MA 02139, USA

**Keywords:** influenza, COVID-19, vaccination, modRNA, immunogenicity, safety, tolerability, clinical trial, combination vaccine

## Abstract

**Background/Objectives**: Vaccination remains an important means of protection against influenza and COVID-19. Administering influenza and COVID-19 vaccines as a combined formulation may be advantageous. **Methods**: This phase 3, randomized study evaluated an investigational combination nucleoside-modified messenger RNA (modRNA) vaccine formulated with monovalent XBB.1.5-adapted BNT162b2 vaccine (BNT162b2) and an investigational influenza modRNA vaccine (hereafter investigational combination modRNA vaccine); we report safety, tolerability, and immunogenicity of the investigational combination modRNA vaccine in healthy 18- to 64-year-olds in two cohorts (2 and 3). **Results**: In Cohort 2, 3127 participants received the investigational combination modRNA vaccine and 1563 received the concomitant licensed quadrivalent influenza vaccine (QIV) with BNT162b2. In Cohort 3, 1189 participants received the investigational combination modRNA vaccine, 605 received QIV, 607 received the investigational influenza modRNA vaccine, and 1191 received BNT162b2. Prespecified noninferiority criteria of the geometric mean ratio of strain-specific hemagglutination inhibition (HAI) or SARS-CoV-2 Omicron XBB.1.5 neutralizing titers or differences in percentages of participants achieving HAI sero-conversion or Omicron XBB.1.5 seroresponse for investigational combination modRNA vaccine to concomitant QIV and BNT162b2 (or to QIV and BNT162b2 each administered alone in Cohort 3) were met for influenza A and SARS-CoV-2 Omicron XBB.1.5 strains but not for the influenza B strain. The investigational combination modRNA vaccine was well tolerated with an acceptable safety profile. **Conclusions**: The single-dose investigational combination influenza plus COVID-19 modRNA vaccine elicited robust immune responses against influenza A and SARS-CoV-2 with acceptable safety. Further work is required to optimize influenza B responses. The modRNA platform offers promise and potential advantages for vaccine development. ClinicalTrials.gov Identifier: NCT06178991 (approval date: 20 December 2023).

## 1. Introduction

Influenza and SARS-CoV-2 infections are leading contributors to infectious respiratory illness burden globally, and the complications of both can range from mild symptoms to severe or even fatal disease, particularly among vulnerable populations [1,2,3,4,5,6]. Approximately a billion cases of seasonal influenza occur globally each year, leading to an estimated 3 to 5 million severe cases and 290,000 to 650,000 deaths [5]. COVID-19 also continues to cause a considerable burden of disease, with ongoing hospitalizations and deaths [7,8]. Therefore, vaccination remains an important means of protecting against the adverse effects of influenza and COVID-19 [4,5].

The nucleoside-modified messenger RNA (modRNA)–based vaccine platform contributed to rapid and large-scale deployment of vaccines during the COVID-19 pandemic [9]. Evidence suggests this platform may also improve vaccine efficacy compared with currently licensed non-modRNA influenza vaccines by providing more precise targeting of vaccine antigens against circulating viral strains together with rapid adaptability [10]. Influenza modRNA vaccines are undergoing clinical development [11].

Influenza and COVID-19 vaccination campaigns currently occur primarily in the late summer and fall to enable protection ahead of the fall and winter viral outbreaks [12]. Administering influenza and COVID-19 vaccines as a combined vaccine formulation would decrease the number of injections, provide added convenience for clinicians and patients, and deliver economic and resource efficiencies, which collectively could contribute to improving vaccination rates [13,14,15,16]. In this context, this phase 3 study evaluated the safety, tolerability, and immunogenicity of an investigational combination influenza plus COVID-19 modRNA vaccine.

## 2. Methods

### 2.1. Study Design

This was a phase 3, observer-blinded, multicohort, randomized study evaluating investigational combination modRNA vaccines; herein we report the results of a combination vaccine formulated with the monovalent BNT162b2 (Omicron XBB.1.5) vaccine (hereafter BNT162b2) and an investigational influenza modRNA vaccine, hereafter collectively referred to as the investigational combination influenza plus COVID-19 modRNA vaccine, in healthy adults 18 through 64 years of age (ClinicalTrials.gov Identifier: NCT06178991; approval date: 20 December 2023). The study evaluated the safety, tolerability, and immunogenicity of the investigational combination influenza plus COVID-19 modRNA vaccine compared with concomitant administration of licensed inactivated quadrivalent influenza vaccine (QIV) with BNT162b2 (hereafter concomitant QIV with BNT162b2) and with standalone administration of QIV or an investigational influenza modRNA vaccine or BNT162b2. The targeted influenza strains for all study vaccines were the seasonal influenza strains recommended by the World Health Organization for cell culture- or recombinant-based vaccines for the Northern Hemisphere’s 2023/2024 influenza season.

Healthy participants or those with pre-existing but stable disease (as defined in the Supplementary Text) could enroll. Individuals vaccinated with any investigational or licensed influenza or COVID-19 vaccine within 6 months before receiving study vaccination were excluded. Full eligibility criteria and details on ethical study conduct are provided in the Supplementary Text.

### 2.2. Randomization and Blinding

Participants were randomized using an interactive response technology system. Treatment assignment information was available only to personnel responsible for investigational product management and administration, including receipt, storage, preparation, dispensing, and dosing activities. Participants were not informed of their assigned randomized intervention, and blinding was maintained for all other sponsor and site personnel, including investigators, study-site staff, sponsor monitoring personnel, safety assessors, and laboratory staff performing serology testing.

### 2.3. Interventions

This study included three cohorts; reported here are the methodology and results for Cohorts 2 and 3, which both evaluated the same investigational combination influenza plus COVID-19 modRNA vaccine. Cohort 1 enrolled approximately 450 participants and evaluated a different investigational combination influenza plus COVID-19 modRNA vaccine (analyses of the data from Cohort 1 are not yet available at the time of this publication). In Cohort 2, approximately 4500 participants were enrolled and randomized in a 2:1 site-based ratio. These participants received either the investigational combination influenza plus COVID-19 modRNA vaccine administered in the right deltoid and placebo (0.9% sodium chloride solution for injection) administered in the left deltoid concurrently or a concomitant administration of licensed QIV (Flucelvax; Seqirus, Inc., Holly Springs, NC, USA) administered in the left deltoid with BNT162b2 administered in the right deltoid.

Cohort 3 was initiated to assess potential interference of the investigational combination influenza plus COVID-19 modRNA vaccine. In this cohort, approximately 3600 participants underwent site-based randomization in a 2:2:1:1 ratio to one of the following four groups: investigational combination influenza plus COVID-19 modRNA vaccine, BNT162b2, licensed QIV, or investigational influenza modRNA vaccine; all vaccines were administered in the right deltoid.

### 2.4. Immunogenicity

The primary immunogenicity objective for Cohort 2 and secondary immunogenicity objective for Cohort 3 was to demonstrate the noninferiority of the immune responses elicited by the investigational combination influenza plus COVID-19 modRNA vaccine measured 4 weeks after vaccination against the influenza and SARS-CoV-2 strains compared with concomitant QIV with BNT162b2 and with standalone QIV, the investigational influenza modRNA vaccine, or BNT162b2. The secondary immunogenicity objective for Cohort 2 was to demonstrate the superiority of immune responses against the influenza strains measured 4 weeks after vaccination with the investigational combination influenza plus COVID-19 modRNA vaccine compared with concomitant QIV with BNT162b2.

For the assessment of primary immunogenicity objectives in Cohort 2, all enrolled participants were tested for SARS-CoV-2 Omicron XBB.1.5–neutralizing titers, and a random subset of approximately 3000 participants (approximately 2000 from the investigational combination influenza plus COVID-19 modRNA vaccine group and approximately 1000 from the concomitant QIV with BNT162b2 group) were tested for hemagglutination inhibition (HAI) titers against matched influenza strains. For the assessment of secondary immunogenicity objectives in Cohort 3, approximately 2400 participants based on their study intervention allocation were tested for HAI titers and Omicron XBB.1.5–neutralizing titers.

Serologic assessments were based on blood samples collected before study vaccine administration and 4 weeks thereafter. Calculated immunogenicity parameters comprised geometric mean antibody titers (GMTs) and the geometric mean fold increases from before receipt of study vaccination to 4 weeks after receipt of study vaccination (i.e., GMFRs) together with the geometric mean ratios (GMRs) between groups. Participants with seroconversion for the influenza strains or with seroresponse for the SARS-CoV-2 strain, both measured as percentages, as well as the corresponding differences in the percentages of responders between groups, were determined. Percentages of participants achieving HAI titers of at least 1:40 were also calculated. Seroconversion to each influenza strain was defined as an HAI titer of less than 1:10 at baseline and of at least 1:40 at 4 weeks after vaccination, or an HAI titer of at least 1:10 at baseline with at least a four-fold rise at 4 weeks after vaccination. Seroresponse against the Omicron XBB.1.5 strain was defined as a minimum four-fold increase relative to baseline; for participants whose baseline titers were below the lower limit of quantification (LLOQ), a post-vaccination value of at least four times the LLOQ was considered indicative of a seroresponse.

### 2.5. Safety

The primary safety endpoints were prespecified local reactions at the injection site of the right deltoid (redness, swelling, injection site pain) and systemic events (fever, vomiting, diarrhea, headache, fatigue, chills, new or worsened muscle pain, new or worsened joint pain), the reports and severity of which (as described in Table S1) were collected daily by all participants in an electronic diary within the 7 days after vaccination (or through to symptom resolution for those symptoms that were ongoing). Primary safety endpoints also included adverse events (AEs) and serious AEs (SAEs) from vaccination through 4 weeks and 6 months after vaccination, respectively. Protocol-defined AEs of special interest (AESIs) were collected for up to 6 months following study vaccination. These included confirmed diagnoses of myocarditis or pericarditis occurring within 6 weeks of study vaccination, as well as influenza, COVID-19, and potential menstrual cycle disturbances reported within 6 months after study vaccination. Protocol-specified evaluations were required for participants reporting symptoms suggestive of myocarditis, pericarditis, or menstrual cycle disturbances.

### 2.6. Statistics

The estimated sample size of up to 8100 participants in Cohorts 2 and 3 was determined to allow for characterization of safety in a large trial population and to provide sufficient statistical power for the evaluation of immunogenicity for primary and secondary objectives.

Analyses of immune responses were performed using the evaluable immunogenicity population. This included participants who were randomized and eligible for the trial, received the vaccine to which they had been assigned, contributed at least a single valid immunogenicity endpoint measurement during the 27- to 42-day post-vaccination window, and remained compliant with the protocol without major deviations.

Hypothesis testing of the objectives for Cohort 2 was evaluated independently from that of Cohort 3 without applying any multiplicity adjustment. Within Cohort 2, the immunogenicity primary objectives and the secondary objective were evaluated sequentially, and the immunogenicity primary estimands were considered coprimary. Hypothesis tests for the immunogenicity primary noninferiority objectives (investigational combination influenza plus COVID-19 modRNA vaccine vs. concomitant QIV with BNT162b2) were performed, with two statistical hypotheses (based on GMR and seroconversion/seroresponse rate difference 4 weeks after vaccination) for each of the targeted influenza and SARS-CoV-2 strains. Each hypothesis was tested at the one-sided alpha level of 0.025. The immunogenicity primary objectives were achieved if noninferiority was met for all statistical hypotheses. Only after the immunogenicity primary noninferiority objectives in Cohort 2 were established would the statistical hypotheses for the secondary objective (i.e., demonstration of superiority of the immune responses measured 4 weeks after vaccination) be tested sequentially in the following order of influenza strains: A/H3N2, A/H1N1, and B/Victoria. The hypothesis was to be tested first based on GMR (investigational combination influenza plus COVID-19 modRNA vaccine group to the comparator group) of HAI titers and then based on the seroconversion rate for each strain; thus, the overall type I error rate within Cohort 2 was fully controlled.

Within Cohort 3, the immunogenicity secondary objectives were evaluated sequentially in the following order: comparing the investigational combination influenza plus COVID-19 modRNA vaccine with QIV for the targeted influenza strains, the investigational combination influenza plus COVID-19 modRNA vaccine with BNT162b2 for the SARS-CoV-2 Omicron XBB.1.5 strain, and the investigational combination influenza plus COVID-19 modRNA vaccine with the investigational influenza modRNA vaccine for the targeted influenza strains. For objectives involving hypotheses for the influenza strains, the hypotheses for all evaluated strains had to be met before assessing the next objective in the sequence; thus, the overall type I error within Cohort 3 was fully controlled.

The GMRs were estimated from analyses conducted on logarithmically transformed immunogenicity data. The exponentiated value of the between-group mean difference on the log scale provided the GMR estimate, and exponentiation of the corresponding 95% confidence limits based on the Student *t* distribution yielded the confidence interval. Between-group differences in responder rates, defined by seroconversion (influenza) or seroresponse (SARS-CoV-2) criteria, were accompanied by determination of two-sided 95% CIs derived using the Miettinen and Nurminen method.

For each strain, noninferiority with respect to GMR was concluded when the lower limit of the two-sided 95% CI exceeded 0.67. In Cohort 2, noninferiority based on percentage of participants with seroconversion (influenza) or seroresponse (SARS-CoV-2) was established when the lower bound of the two-sided 95% CI for the between-group difference in responder rates was greater than −10%. Superiority in Cohort 2 was demonstrated when the lower limit of the two-sided 95% CI was above 1.0 for the GMR or above 0% for the difference in seroconversion rates between groups.

The GMTs were derived by first applying a logarithmic transformation to assay values, calculating the mean of the transformed data, and then back-transforming the result to the original scale by exponentiation. The two-sided 95% CIs were determined by computing intervals on the log-transformed data using the Student *t* distribution and subsequently exponentiating the confidence limits. The GMFRs were calculated from the mean difference in logarithmically transformed assay results at two time points (later time point minus earlier time point), followed by exponentiation of the mean. Corresponding two-sided 95% CIs for GMFRs were constructed by applying the Student *t* distribution to the mean difference on the logarithm scale and transforming the resulting confidence limits to the original scale through exponentiation.

Percentage-based immune response endpoints, including participants achieving seroconversion, seroresponse, and an HAI antibody titer of at least 1:40, were evaluated within each study intervention group. Associated two-sided 95% CIs were calculated using the Clopper–Pearson method for each vaccine group.

The safety population consisted of all study intervention recipients and served as the basis for safety analyses. For binary safety endpoints such as reactogenicity events and AEs, summaries included percentages of participants, the associated numerator (*n*) and denominator (N) values used to derive those percentages, together with two-sided 95% CIs, where applicable, which were computed using the Clopper–Pearson method.

## 3. Results

### 3.1. Participants

**Figure 1:**
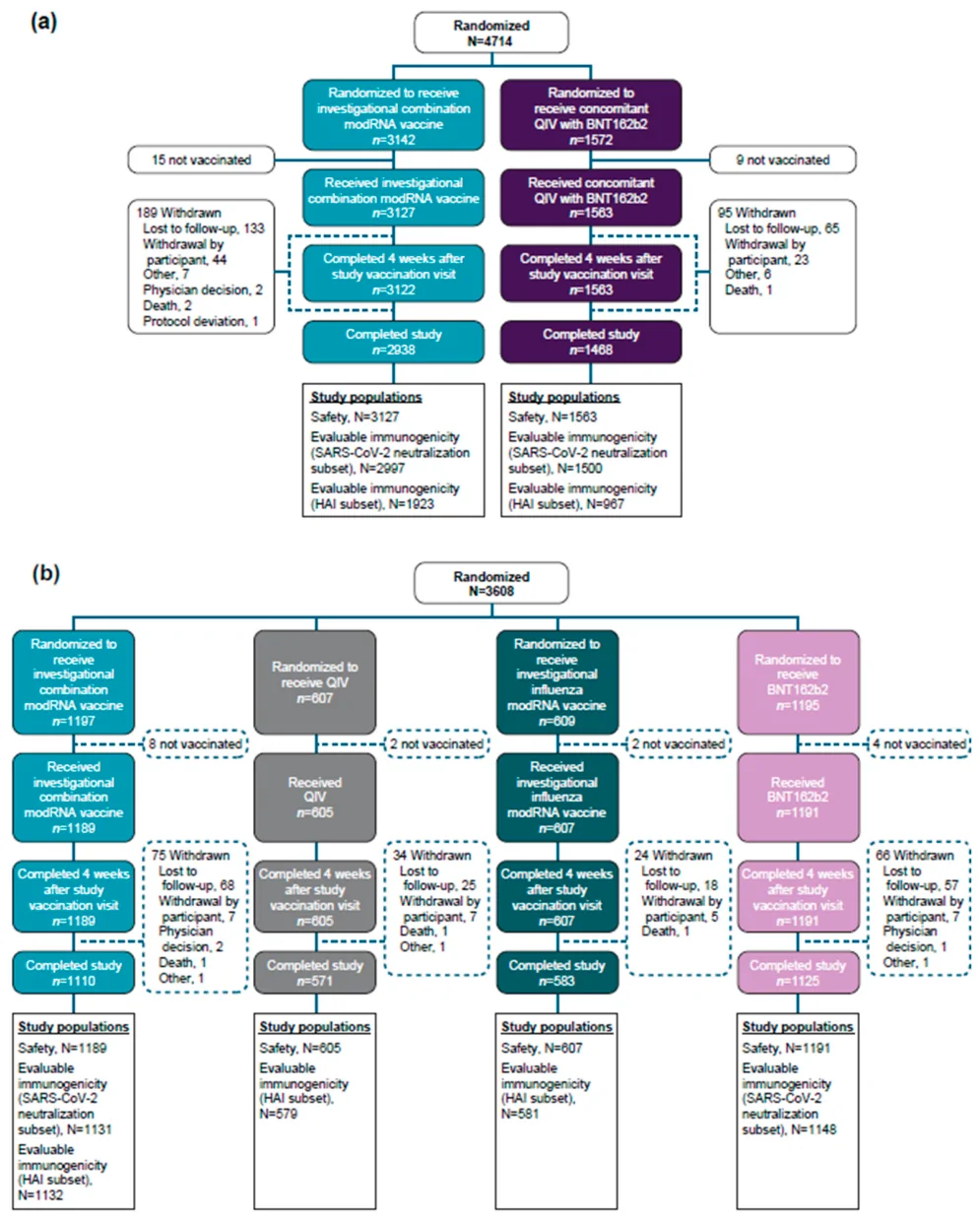
Participant disposition and analysis populations in (**a**) Cohort 2 and (**b**) Cohort 3. BNT162b2, monovalent BNT162b2 (Omicron XBB.1.5) vaccine; HAI, hemagglutination inhibition; investigational combination modRNA vaccine, investigational combination influenza plus COVID-19 modRNA vaccine; modRNA, nucleoside-modified messenger RNA; QIV, licensed quadrivalent influenza vaccine.

This study was conducted between 30 January 2024 and 26 November 2024 at 106 sites in the United States. In Cohort 2, 3127 participants received the investigational combination influenza plus COVID-19 modRNA vaccine and 1563 participants received concomitant QIV with BNT162b2; most participants completed at least 6 months of follow-up after vaccination (investigational combination influenza plus COVID-19 modRNA vaccine, *n* = 3031; concomitant QIV with BNT162b2, *n* = 1514; Figure 1a). In Cohort 3, 1189 participants received the investigational combination influenza plus COVID-19 modRNA vaccine, 605 received QIV, 607 received the investigational influenza modRNA vaccine, and 1191 received BNT162b2; most participants completed at least 6 months of follow-up after vaccination (investigational combination influenza plus COVID-19 modRNA vaccine, *n* = 1159; QIV, *n* = 595; investigational influenza modRNA vaccine, *n* = 597; BNT162b2, *n* = 1171; Figure 1b).

Demographic and baseline characteristics of Cohorts 2 and 3 (Table 1 and Table 2, respectively) were well balanced across the vaccine groups. Overall in Cohort 2, 55.2% of participants were female, the median age at vaccination was 45 years, and the majority of participants were White (67.3%) and non-Hispanic/non-Latino (68.2%). Most participants had evidence of prior SARS-CoV-2 infection (89.3%), 29.9% had received an influenza vaccination within 3 years of enrollment, and 74.8% had previously received at least 1 dose of a COVID-19 vaccine. Overall in Cohort 3, 55.8% of participants were female, the median age at vaccination was 46 years, and the majority of participants were White (70.0%) and non-Hispanic/non-Latino (61.3%). Most participants had evidence of previous SARS-CoV-2 infection (89.6%), 33.2% had received an influenza vaccination within 3 years of enrollment, and 67.9% had received at least one previous dose of a COVID-19 vaccine.

### 3.2. Immunogenicity

**Figure 2:**
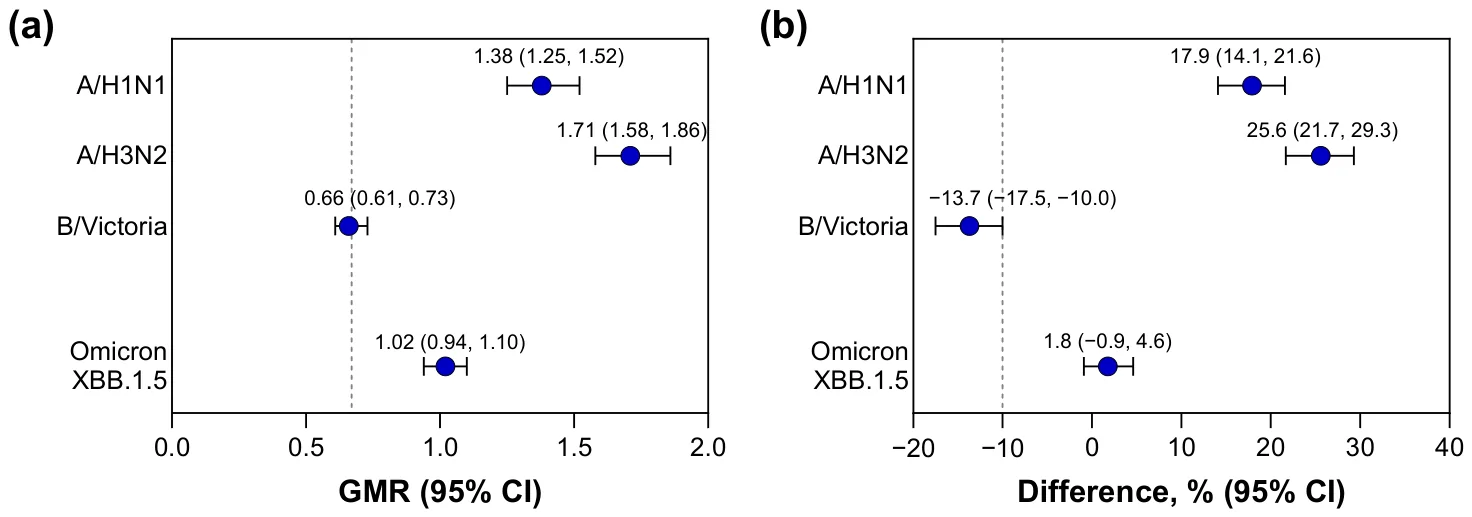
Forest plots of (**a**) GMR of strain-specific HAI and SARS-CoV-2 Omicron XBB.1.5 neutralizing titers and (**b**) difference in percentage of participants achieving HAI seroconversion or SARS-CoV-2 Omicron XBB.1.5 seroresponse for investigational combination influenza plus COVID-19 mod-RNA vaccine to the comparator group of concomitant QIV with BNT162b2 in Cohort 2. Details regarding study population and statistical assessments are included in the Methods. The dashed vertical line in panel (**a**) is the 1.5-fold criterion to declare noninferiority (lower bound of the two-sided 95% CI > 0.67). The dashed line in panel (**b**) is the noninferiority margin, which was declared if the lower limit of the two-sided 95% CI for the difference in percentage of participants achieving seroconversion or seroresponse was greater than −10%. BNT162b2, monovalent BNT162b2 (Omicron XBB.1.5) vaccine; GMR, geometric mean ratio; HAI, hemagglutination inhibition; modRNA, nucleoside-modified messenger RNA; QIV, quadrivalent influenza vaccine.

In Cohort 2, the prespecified criterion to declare noninferiority of the GMR of strain-specific HAI titers or SARS-CoV-2 Omicron XBB.1.5 neutralizing titers for the investigational combination influenza plus COVID-19 modRNA vaccine versus concomitant QIV with BNT162b2 (i.e., the 1.5-fold criterion of the lower bound of the two-sided 95% CI greater than 0.67) was met for the A/H1N1, A/H3N2, and SARS-CoV-2 Omicron XBB.1.5 strains but not for the influenza B/Victoria strain (Figure 2a). The prespecified criterion to declare noninferiority of the difference in percentage of participants achieving HAI seroconversion or Omicron XBB.1.5 seroresponse for the investigational combination influenza plus COVID-19 modRNA vaccine versus concomitant QIV with BNT162b2 was met for the A/H1N1, A/H3N2, and SARS-CoV-2 Omicron XBB.1.5 strains but not for the influenza B/Victoria strain (Figure 2b). Because noninferiority was not demonstrated for all targeted strains evaluated, superiority was not formally assessed. However, the lower bounds of the two-sided 95% CIs for GMRs would have met superiority for the investigational combination influenza plus COVID-19 modRNA vaccine compared with concomitant QIV with BNT162b2 for the A/H1N1 and A/H3N2 strains, which were 1.25 and 1.58, respectively, consistent with the superiority criterion (lower bound of the two-sided 95% CI for GMR greater than 1). Similarly, the lower bound of the two-sided 95% CI for difference in percentages of participants achieving HAI seroconversion of the investigational combination influenza plus COVID-19 modRNA vaccine compared with concomitant QIV with BNT162b2 for the A/H1N1 and A/H3N2 strains was 14.1% and 21.7%, respectively, which is consistent with the superiority criterion (lower bound of the two-sided 95% CI for difference in seroconversion rate greater than 0).

**Figure 3:**
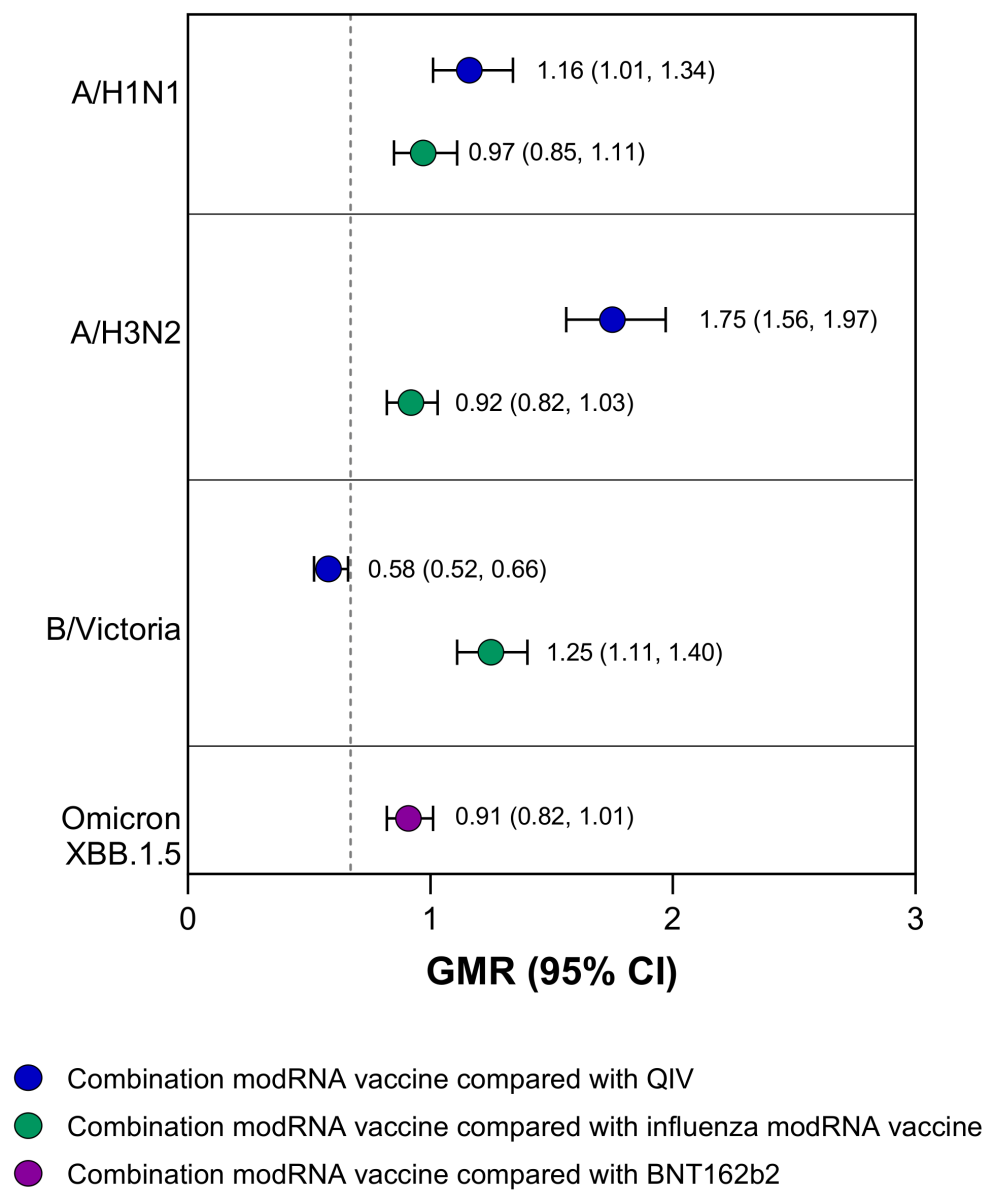
Forest plot of GMR of strain-specific HAI and SARS-CoV-2 Omicron XBB.1.5 neutralizing titers comparing the investigational combination influenza plus COVID-19 modRNA vaccine with the comparator QIV, investigational influenza modRNA vaccine, and BNT162b2 groups in Cohort 3. Details regarding study population and statistical assessments are included in the Methods. The dashed vertical line is the 1.5-fold criterion to declare noninferiority (lower bound of the two-sided 95% CI greater than 0.67). BNT162b2, monovalent BNT162b2 (Omicron XBB.1.5) vaccine; combination modRNA vaccine, investigational combination influenza plus COVID-19 modRNA vaccine; GMR, geometric mean ratio; HAI, hemagglutination inhibition; influenza modRNA vaccine, investigational influenza modRNA vaccine; modRNA, nucleoside-modified messenger RNA; QIV, quadrivalent influenza vaccine.

Cohort 3 was initiated to compare the investigational combination influenza plus COVID-19 modRNA vaccine with standalone administration of QIV or an investigational influenza modRNA vaccine or BNT162b2. In Cohort 3, the prespecified criterion to declare noninferiority of the GMR of the investigational combination influenza plus COVID-19 modRNA vaccine to licensed QIV (lower bound of the two-sided 95% CI greater than 0.67) was met and was greater than 1 for both influenza A strains but not for the B/Victoria strain (Figure 3). Sequential hypothesis testing of noninferiority of the investigational combination influenza plus COVID-19 modRNA vaccine versus BNT162b2 for the SARS-CoV-2 Omicron XBB.1.5 strain and versus the investigational influenza modRNA vaccine for the targeted influenza strains could not proceed for the other comparisons. However, the lower bounds of the two-sided 95% CIs for the GMRs of the investigational combination influenza plus COVID-19 modRNA vaccine compared with BNT162b2 for the SARS-CoV-2 Omicron XBB.1.5 strain and compared with the investigational influenza modRNA vaccine for all influenza strains were at least 0.82, which was above the noninferiority criterion.

**Figure 4:**
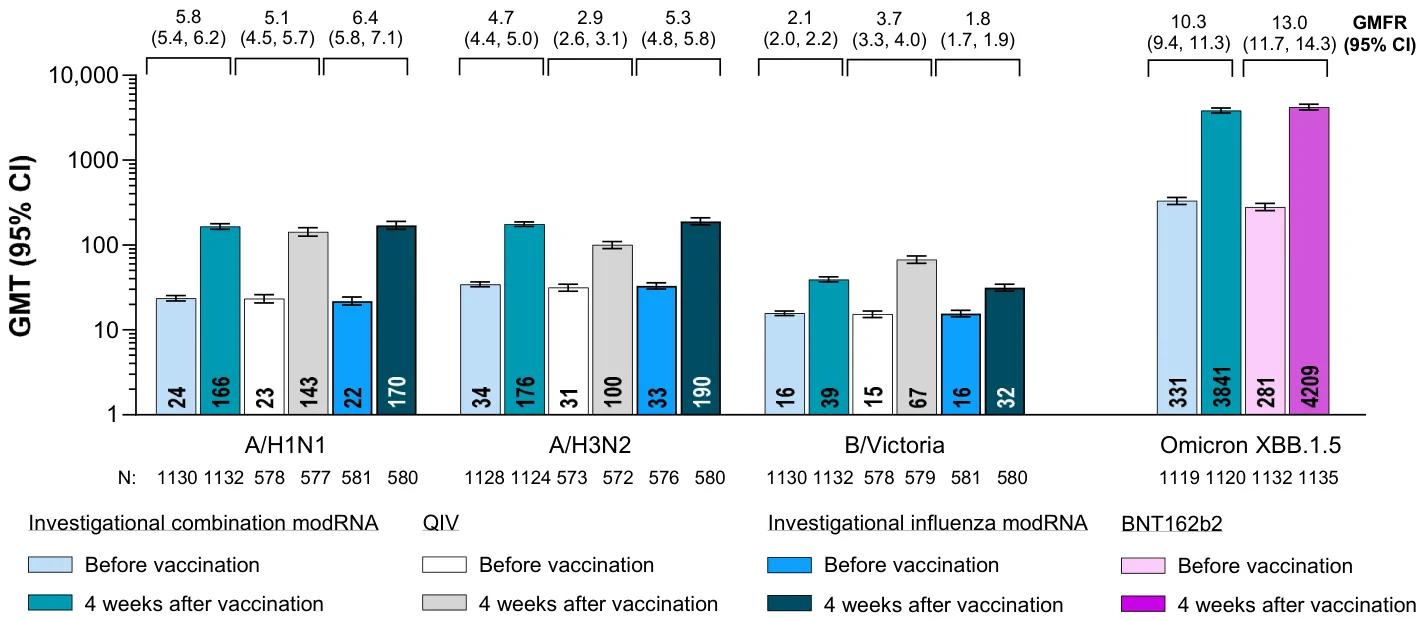
GMTs and GMFRs of strain-specific HAI titers and SARS-CoV-2 Omicron XBB.1.5 neutralizing titers in Cohort 3. BNT162b2, monovalent BNT162b2 (Omicron XBB.1.5) vaccine; GMFR, geometric mean fold rise from baseline to 4 weeks after vaccination; GMT, geometric mean titer; HAI, hemagglutination inhibition; investigational combination modRNA, investigational combination influenza plus COVID-19 modRNA vaccine; modRNA, nucleoside-modified messenger RNA; QIV, quadrivalent influenza vaccine.

The GMTs and GMFRs of strain-specific HAI titers and SARS-CoV-2 Omicron XBB.1.5 neutralizing titers in Cohort 3 are shown in Figure 4. Baseline HAI GMTs were similar across strains in the investigational combination influenza plus COVID-19 mod-RNA vaccine, QIV, and investigational influenza modRNA vaccine groups, ranging from 21.8 to 23.6, 31.4 to 34.3, and 15.3 to 15.7 for the A/H1N1, A/H3N2, and B/Victoria strains, respectively. GMFRs from baseline to 4 weeks after vaccination in the investigational combination influenza plus COVID-19 modRNA vaccine and investigational influenza modRNA vaccine groups were greater than 4 for both influenza A strains but reached that level only in the QIV group for the A/H1N1 strain. GMFRs for the B/Victoria strain were less than 4 for all three vaccine groups. Baseline neutralizing GMTs against the Omicron XBB.1.5 strain were similar in the investigational combination influenza plus COVID-19 modRNA vaccine and BNT162b2 groups (331.4 vs. 281.0), and GMFRs from baseline to 4 weeks after vaccination were greater than 10 for both groups.

**Figure 5:**
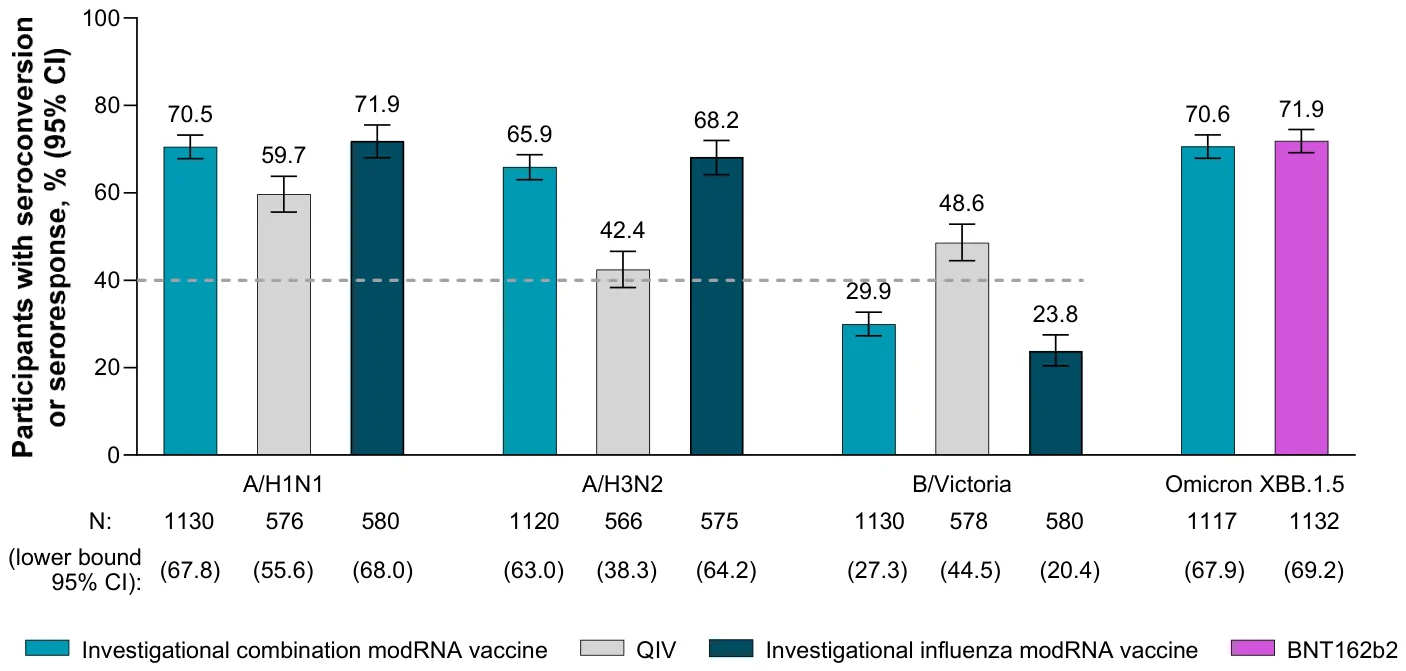
Percentage of participants from Cohort 3 with seroconversion (influenza strains) or sero-response (SARS-CoV-2 strain) measured 4 weeks after vaccination. The horizontal dashed line indicates the CBER/CHMP threshold for acceptable seroconversion for influenza strains of a lower-bound 95% CI of 40%. BNT162b2, monovalent BNT162b2 (Omicron XBB.1.5) vaccine; CBER, US Food and Drug Administration’s Center for Biologics Evaluation and Research; CHMP, European Medicines Agency’s Committee for Medicinal Products for Human Use; HAI, hemagglutination inhibition; investigational combination modRNA vaccine, investigational combination influenza plus COVID-19 modRNA vaccine; modRNA, nucleoside-modified messenger RNA; QIV, quadrivalent influenza vaccine.

The percentages of participants from Cohort 3 with HAI seroconversion and SARS-CoV-2 seroresponse are shown in Figure 5. The lower bound of the two-sided 95% CI for HAI seroconversion rates in the investigational combination influenza plus COVID-19 modRNA vaccine group and investigational influenza modRNA vaccine group met the US Food and Drug Administration’s Center for Biologics Evaluation and Research (CBER) and the European Medicines Agency’s Committee for Medicinal Products for Human Use (CHMP) defined threshold for influenza strains of at least 40% for A/H1N1 (lower bound 95% CI of 67.8% and 68.0%, respectively) and A/H3N2 (lower bound 95% CI 63.0% and 64.2%) but did not meet this threshold for B/Victoria (lower bound 95% CI 27.3% and 20.4%). In the QIV group, the two-sided 95% CI lower bound threshold was met for A/H1N1 (lower bound 95% CI 55.6%) and B/Victoria (lower bound 95% CI 44.5%) but not for A/H3N2 (lower bound 95% CI 38.3%). SARS-CoV-2 seroresponse rates were greater than 70% in the investigational combination influenza plus COVID-19 modRNA vaccine and BNT162b2 groups.

The percentages of participants with HAI titers of at least 1:40 before and 4 weeks after vaccination are shown in Figure S1. The lower bound of the two-sided 95% CI for the percentage of participants with HAI titers of at least 1:40 in all vaccine groups (i.e., investigational combination influenza plus COVID-19 modRNA vaccine, QIV, and investigational influenza modRNA vaccine) met the CBER/CHMP defined threshold of at least 70% for A/H1N1 and A/H3N2 strains, but only the QIV group met this threshold for the B/Victoria strain.

### 3.3. Safety

**Figure 6:**
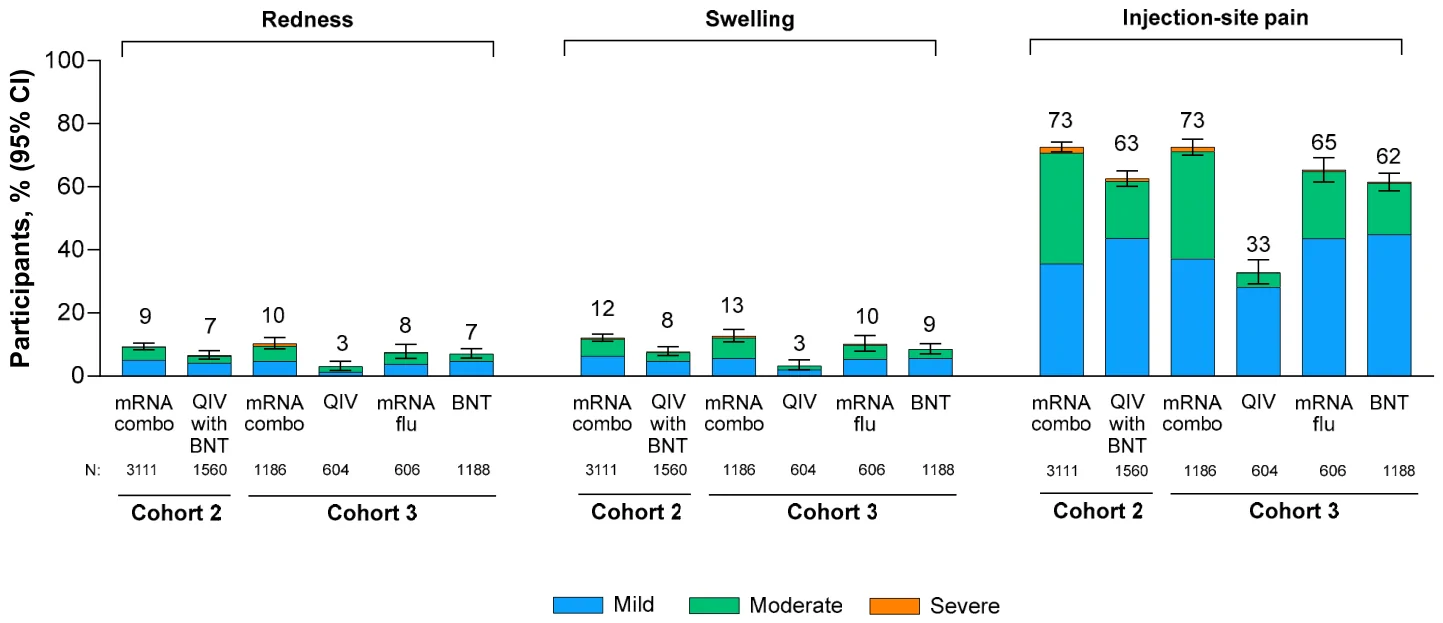
Percentage of participants in Cohorts 2 and 3 reporting local reactions within 7 days after vaccination by severity grade. Values displayed above each bar indicate the overall percentage of participants who reported the corresponding local reaction. Local reactions are those occurring in the right deltoid only (for the QIV with BNT group, QIV was administered to the left deltoid and BNT was administered to the right deltoid). BNT, monovalent BNT162b2 (Omicron XBB.1.5) vaccine; modRNA, nucleoside-modified messenger RNA; mRNA combo, investigational combination influenza plus COVID-19 modRNA vaccine; mRNA flu, investigational influenza modRNA vaccine; QIV, quadrivalent influenza vaccine; QIV with BNT, concomitant quadrivalent influenza vaccine given with monovalent BNT162b2 (Omicron XBB.1.5) vaccine.

Across both cohorts, local reactions within 7 days of vaccination were reported less frequently in participants who received QIV (34.8%) than in participants who received BNT162b2 (62.5–63.5%) or investigational combination influenza plus COVID-19 mod-RNA vaccine (73.4% in the Cohort 2 group as well as in the Cohort 3 group) or the investigational influenza modRNA vaccine (66.3%). The most frequently reported local reaction across all vaccine groups was injection site pain, which was reported more frequently in the investigational combination influenza plus COVID-19 modRNA vaccine (72.6% in the Cohort 2 group as well as in the Cohort 3 group), investigational influenza modRNA vaccine (65.3%), and BNT162b2 groups (61.6–62.6%) than in the QIV group (32.9%; Figure 6). The majority of local reactions were mild or moderate in severity. Severe local reactions were less common, occurring in ≤1.9% of participants, and no grade 4 local reactions were reported. Across vaccine groups, the median onset day of local reactions was between the day of vaccination and 1 day after vaccination, and median duration was 1 to 2 days; onset and duration of local reactions were similar between groups (Table S2).

**Figure 7:**
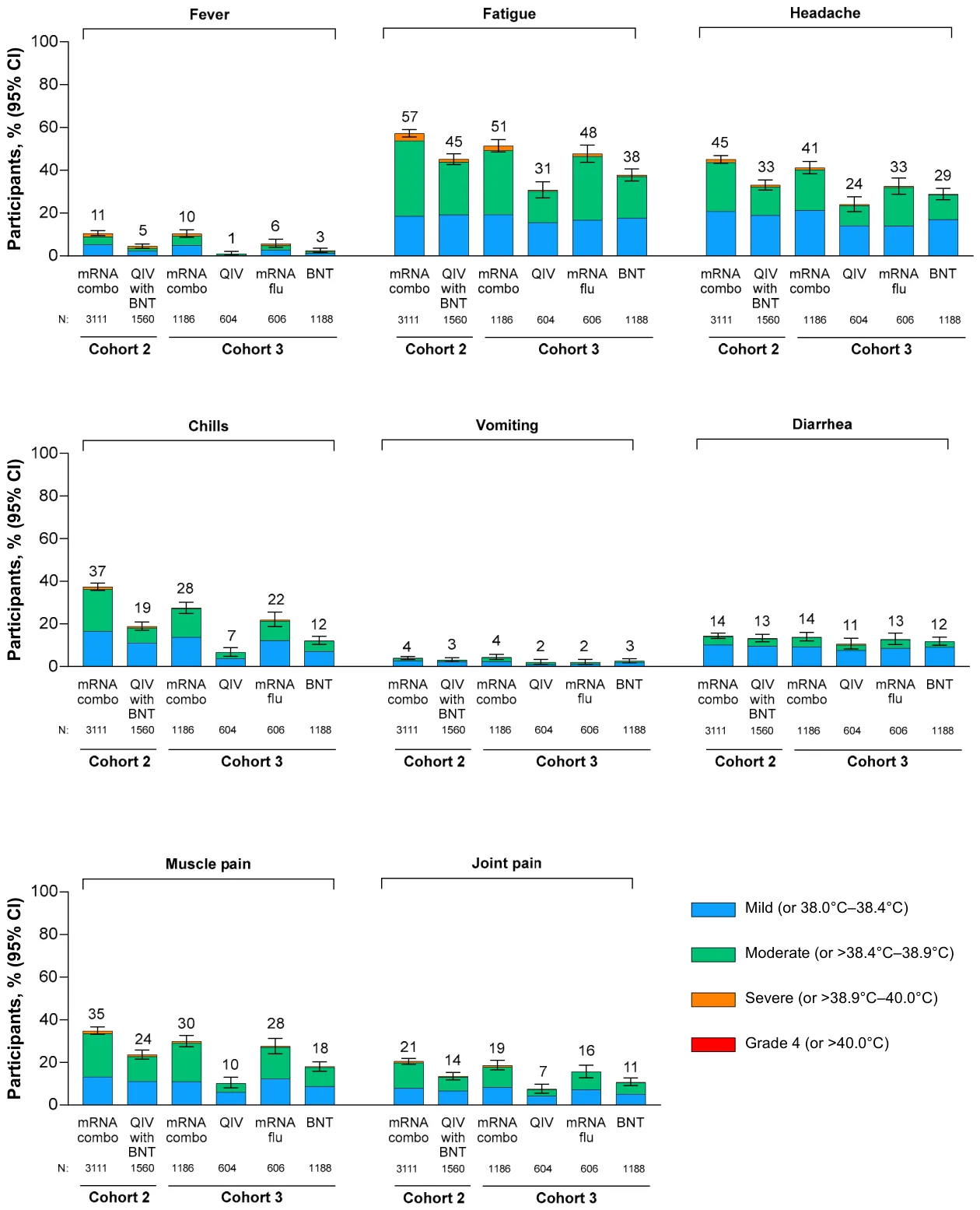
Percentage of participants in Cohorts 2 and 3 reporting systemic events within 7 days after vaccination by severity grade. Values displayed above each bar indicate the overall percentage of participants who reported the corresponding systemic event. BNT, monovalent BNT162b2 (Omicron XBB.1.5) vaccine; modRNA, nucleoside-modified messenger RNA; mRNA combo, investigational combination influenza plus COVID-19 modRNA vaccine; mRNA flu, investigational influenza modRNA vaccine; QIV, quadrivalent influenza vaccine; QIV with BNT, concomitant quadrivalent influenza vaccine given with monovalent BNT162b2 (Omicron XBB.1.5) vaccine.

Across both cohorts, systemic events within 7 days of vaccination were reported less frequently in QIV (43.9%) recipients than in investigational combination influenza plus COVID-19 modRNA vaccine (66.7–70.8%), concomitant QIV with BNT162b2 (59.9%), investigational influenza modRNA vaccine (61.1%), or BNT162b2 (52.1%) recipients. The most frequently reported systemic events across vaccine groups were fatigue, headache, new or worsened muscle pain, and chills (Figure 7). Frequently reported systemic events in the investigational modRNA vaccine groups tended to be reported at somewhat higher frequencies than in the comparator groups. Most systemic events were mild or moderate in severity; severe systemic events were reported in no more than 3.4% of participants. Three participants experienced fever of higher than 40.0 °C (investigational combination influenza plus COVID-19 modRNA vaccine, *n* = 2 [<0.1%]; concomitant QIV with BNT162b2, *n* = 1 [<0.1%]). No grade 4 systemic events were reported. Across vaccine groups, the median onset day of systemic events was between 1 and 2 days after vaccination, and median duration was 1 to 2 days; onset and duration of systemic events were similar between groups (Table S2).

**Figure 8:**
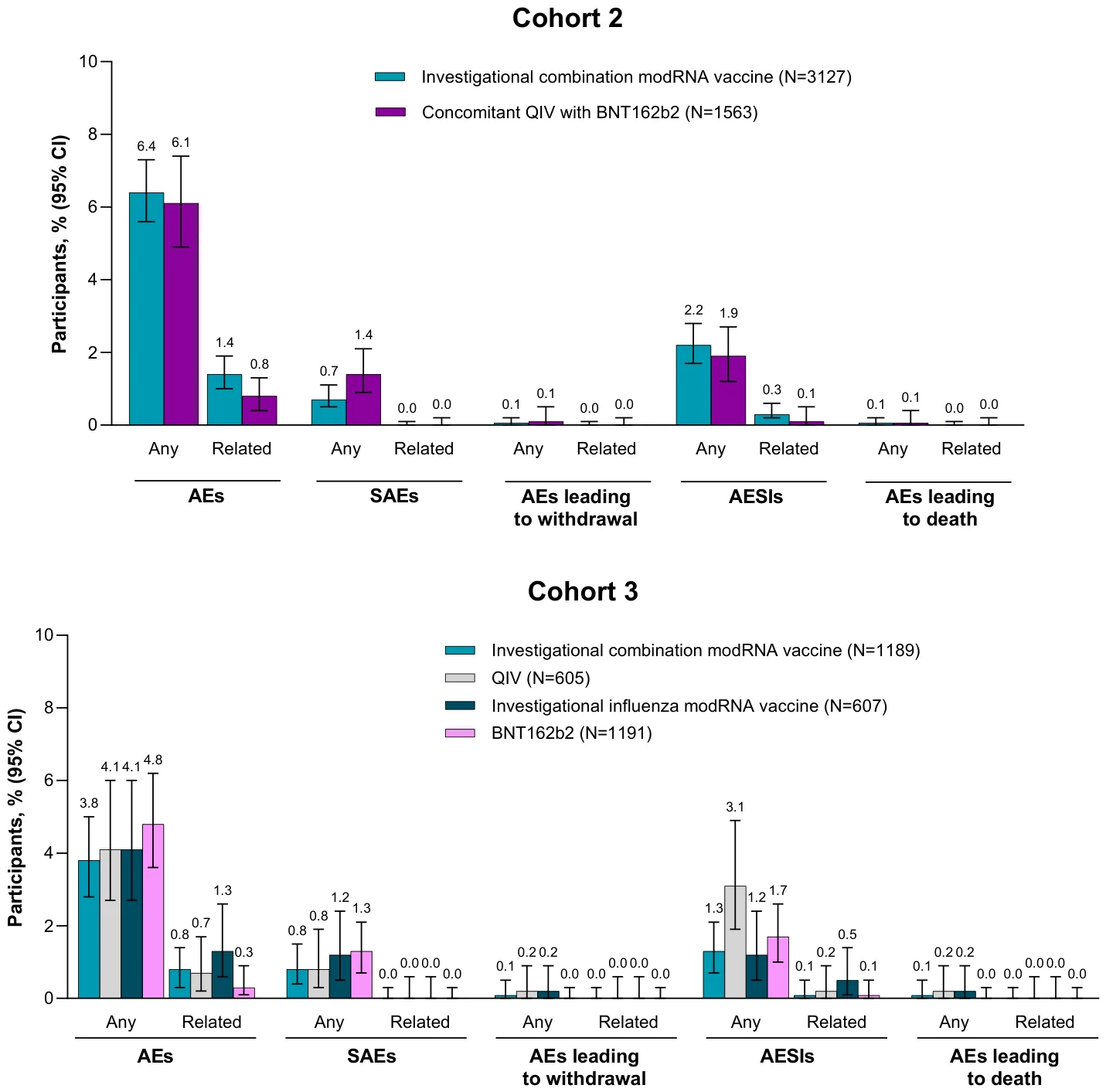
Adverse events in Cohorts 2 and 3. AEs are through 4 weeks after vaccination, and the remaining endpoints are through 6 months after vaccination. AE, adverse event; AESI, adverse event of special interest; BNT162b2, monovalent BNT162b2 (Omicron XBB.1.5) vaccine; investigational combination modRNA vaccine, investigational combination influenza plus COVID-19 modRNA vaccine; modRNA, nucleoside-modified messenger RNA; QIV, quadrivalent influenza vaccine; SAE, serious adverse event.

Across study groups and cohorts, AEs through 4 weeks after vaccination were reported by 3.8% to 6.4% of participants; no more than 1.4% of participants in any vaccine group experienced AEs that were considered related to study vaccination by the investigator (Figure 8). In Cohort 2, lymphadenopathy regardless of causality was reported by nine participants (0.3%) who received investigational combination influenza plus COVID-19 modRNA vaccine and four participants (0.3%) who received concomitant QIV with BNT162b2 and, in Cohort 3, by four participants (0.3%) who received investigational combination influenza plus COVID-19 modRNA vaccine. Through 6 months after vaccination, AEs leading to study withdrawal were reported in <0.1% of participants who received the investigational combination influenza plus COVID-19 modRNA vaccine in Cohorts 2 and 3, and in no more than 0.2% of participants in any vaccine group (Figure 8). Among participants who withdrew from the study because of an AE, none of the events were assessed as study vaccine-related by the investigator. Low percentages of participants reported SAEs during the 6 months following receipt of study intervention, with similar percentages of participants reporting SAEs in each vaccine group (ranging from 0.7% in the Cohort 2 investigational combination influenza plus COVID-19 modRNA vaccine group to 1.4% in the concomitant QIV with BNT162b2 group); none were considered related to study vaccination by the investigator (Table S3). Six deaths in total occurred, with three deaths in Cohort 2 (investigational combination influenza plus COVID-19 modRNA vaccine, *n* = 2 [<0.1%]; concomitant QIV with BNT162b2, *n* = 1 [<0.1%]) and three deaths in Cohort 3 (investigational combination influenza plus COVID-19 modRNA vaccine, *n* = 1 [<0.1%]; QIV, *n* = 1 [0.2%]; investigational influenza modRNA vaccine, *n* = 1 [0.2%]), and all were assessed as unrelated to study vaccination by the investigator.

Across both cohorts, no confirmed cases of myocarditis or pericarditis were reported. Cases of confirmed COVID-19 (≤2.0%) and influenza (≤0.3%) were observed at similar frequencies between groups (Table S4). Menstrual cycle disturbances were reported in up to 0.7% of participants in Cohort 2 (investigational combination influenza plus COVID-19 modRNA vaccine, *n* = 23 [0.7%]; concomitant QIV with BNT162b2, *n* = 3 [0.2%]) and in up to 1.2% of participants in Cohort 3 (investigational combination influenza plus COVID-19 modRNA vaccine, *n* = 2 [0.2%]; QIV, *n* = 7 [1.2%]; investigational influenza modRNA vaccine, *n* = 3 [0.5%]; BNT162b2, *n* = 5 [0.4%]).

## 4. Discussion

This phase 3 study in adults 18 through 64 years of age evaluated an investigational combination influenza plus COVID-19 modRNA vaccine compared with concomitant administration of a licensed quadrivalent influenza vaccine with the licensed COVID-19 BNT162b2 (Omicron XBB.1) modRNA vaccine. The study also compared the investigational combination influenza plus COVID-19 modRNA vaccine with standalone administration of the licensed influenza vaccine, an investigational influenza modRNA vaccine, and licensed COVID-19 BNT162b2 (Omicron XBB.1) modRNA vaccine. Administration of more than a single vaccine through multi-vaccine formulations is common in pediatric immunization programs and for travelers, but it is less well established in other adult populations [17]. The administration of immunogenic and well-tolerated combination modRNA vaccines against influenza and COVID-19 affords the potential benefits of increased vaccine uptake, improved protection against these infectious diseases, and decreased resource utilization [17]. Vaccine recommendations for these pathogens in at-risk individuals also commonly coincide [12]. Collectively, because adult populations are at continued risk of adverse outcomes from both influenza and COVID-19, receipt of a combined modRNA vaccine in at-risk adult populations has likely public health benefits [1,2,3,4,5,6].

The results of our study support that influenza responses following vaccination with influenza-encoding modRNA-containing vaccines show antibody responses to influenza A/H3N2 and A/H1N1 strains that are similar to or higher than those measured in individuals receiving the licensed influenza vaccine, while influenza B responses trended lower. Additionally, influenza B responses were reduced overall in both the investigational mod-RNA vaccine groups and in the licensed influenza vaccine group compared with responses against influenza A strains. The finding that influenza B responses were lower compared with influenza A responses across investigational and licensed influenza vaccine groups has been observed previously [11,18,19].

Cohort 3 assessed the potential for immune interference between the influenza and COVID-19 components of the investigational combination influenza plus COVID-19 mod-RNA vaccine. Consistent with the observations from Cohort 2, the trend toward higher influenza A responses in the investigational combination influenza plus COVID-19 mod-RNA vaccine group was maintained, whereas influenza B responses were lower when compared against licensed QIV. When compared with the standalone investigational influenza modRNA vaccine, the immune responses elicited by the investigational combination influenza plus COVID-19 modRNA vaccine were similar for all vaccine-matched influenza strains. The investigational combination influenza plus COVID-19 modRNA vaccine also performed favorably against the licensed COVID-19 modRNA vaccine. Collectively, this suggests minimal interference in immune responses between the modRNA influenza and COVID-19 vaccines. These results are encouraging, suggesting that investigational combination modRNA vaccines elicit HAI responses that are overall favorable and comparable to those observed with concomitant administration of licensed QIV and licensed QIV-only vaccine administration, with continued work optimizing influenza B responses. The reason for the observed lower influenza B compared with influenza A strain responses observed with influenza vaccines is unknown but could include history of immune exposure, assay limitations, and influenza B immunogenic properties [20].

Safety analyses from the study’s two cohorts showed the investigational combination influenza plus COVID-19 modRNA vaccine was well tolerated. Local reactions and systemic events were reported less frequently after QIV than after modRNA influenza or COVID-19 vaccine receipt either individually or in a combination vaccine. In all study groups, including the investigational combination influenza plus COVID-19 modRNA vaccine group, the majority of reactogenicity events were mild or moderate in severity and of early onset (between the day of vaccination and 2 days after vaccination) and of short duration (between 1 and 2 days in all vaccine groups), with both onset and duration of reactogenicity events similar between groups.

No new or unanticipated safety concerns were identified with the investigational combination influenza plus COVID-19 modRNA vaccine or in any of the comparator vaccine groups. AEs through 4 weeks after vaccination were reported in 3.8% to 6.4% of participants across vaccine groups, with no more than 1.4% of participants having AEs considered to be vaccine-related. Lymphadenopathy was reported in 0.3% of participants who received investigational combination influenza plus COVID-19 modRNA vaccine both in Cohort 2 and in Cohort 3 and in 0.3% of those who received concomitant QIV with BNT162b2. No participant withdrew from the study because of an AE considered study vaccine-related by the investigator. Through 6 months after vaccination, serious adverse events were infrequent and occurred with similar frequency across vaccine groups (ranging from 0.7 to 0.8% in the investigational combination influenza plus COVID-19 mod-RNA vaccine groups to 1.4% in the concomitant influenza with COVID-19 vaccine group), with none considered to be vaccine-related. AESIs were generally balanced across vaccine groups, and there were no confirmed cases of myocarditis or pericarditis. The AE profile of the study vaccines generally reflected unrelated infections, illnesses, and conditions that may be expected in these age groups.

Limitations of the study include that recruitment occurred only in the United States, possibly limiting generalizability to other regions. Similarly, although the findings support the potential of combined modRNA vaccination strategies, further characterization in other populations and age groups is not being pursued at this time with this vaccine candidate. Additionally, the 4-week follow-up for immunogenicity means that durability of immune responses could not be determined.

## 5. Conclusions

In conclusion, a single-dose investigational combination influenza plus COVID-19 modRNA vaccine elicited robust immune responses to influenza A and SARS-CoV-2 strains and had an acceptable safety profile in a large phase 3 data set. Although further clinical investigations are anticipated, these data provide a strong foundation for the mod-RNA platform, which shows substantial promise and potential advantages in the development of influenza and combination vaccines.

## Figures and Tables

**Table 1:** Baseline participant demographic characteristics in Cohort 2.

	Investigational Combination Influenza Plus COVID-19 modRNA Vaccine (N = 3127)	Concomitant QIV with BNT162b2 (N = 1563)
Sex, *n* (%)		
Male	1387 (44.4)	715 (45.7)
Female	1740 (55.6)	848 (54.3)
Race, *n* (%)		
White	2106 (67.3)	1051 (67.2)
Black	812 (26.0)	392 (25.1)
Asian	111 (3.5)	58 (3.7)
American Indian or Alaska Native	22 (0.7)	17 (1.1)
Native Hawaiian or Other Pacific Islander	12 (0.4)	2 (0.1)
Multiracial	32 (1.0)	24 (1.5)
Not reported	32 (1.0)	19 (1.2)
Ethnicity, *n* (%)		
Hispanic/Latino	966 (30.9)	491 (31.4)
Non-Hispanic/non-Latino	2145 (68.6)	1055 (67.5)
Not reported	16 (0.5)	17 (1.1)
Age at vaccination, years		
Mean (SD)	44.0 (12.9)	44.2 (12.5)
Median (range)	45 (18–64)	46 (18–64)
Baseline SARS-CoV-2 status, ^a^ *n* (%)		
Positive	2803 (89.6)	1386 (88.7)
Negative	316 (10.1)	174 (11.1)
Missing	8 (0.3)	3 (0.2)
Received previous influenza vaccination, ^b^ *n* (%)	926 (29.6)	475 (30.4)
Number of previous influenza vaccinations, ^b^ *n* (%)		
1	733 (23.4)	386 (24.7)
2	179 (5.7)	81 (5.2)
3	14 (0.4)	8 (0.5)
Received previous COVID-19 vaccination, *n* (%)	2319 (74.2)	1187 (75.9)
Number of previous COVID-19 vaccinations, *n* (%)		
1	314 (10.0)	191 (12.2)
2	847 (27.1)	405 (25.9)
3	763 (24.4)	396 (25.3)
4	323 (10.3)	154 (9.9)
5	66 (2.1)	36 (2.3)
6	6 (0.2)	5 (0.3)

BNT162b2, monovalent BNT162b2 (Omicron XBB.1.5) vaccine; modRNA, nucleoside-modified messenger RNA; QIV, licensed quadrivalent influenza vaccine. ^a^ Baseline SARS-CoV-2 positivity required a positive N-binding assay result at baseline or a reported history of COVID-19. ^b^ In the previous 3 years only.

**Table 2:** Baseline participant demographic characteristics in Cohort 3.

	Investigational Combination Influenza Plus COVID-19 modRNA Vaccine (N = 1189)	QIV (N = 605)	Investigational Influenza modRNA Vaccine (N = 607)	BNT162b2 (N = 1191)
Sex, *n* (%)				
Male	535 (45.0)	269 (44.5)	275 (45.3)	508 (42.7)
Female	654 (55.0)	336 (55.5)	332 (54.7)	683 (57.3)
Race, *n* (%)				
White	830 (69.8)	425 (70.2)	419 (69.0)	842 (70.7)
Black	277 (23.3)	143 (23.6)	157 (25.9)	295 (24.8)
Asian	35 (2.9)	18 (3.0)	20 (3.3)	34 (2.9)
American Indian or Alaska Native	13 (1.1)	4 (0.7)	2 (0.3)	4 (0.3)
Native Hawaiian or Other Pacific Islander	9 (0.8)	2 (0.3)	2 (0.3)	3 (0.3)
Multiracial	15 (1.3)	7 (1.2)	2 (0.3)	7 (0.6)
Not reported	10 (0.8)	6 (1.0)	5 (0.8)	6 (0.5)
Ethnicity, *n* (%)				
Hispanic/Latino	457 (38.4)	224 (37.0)	225 (37.1)	435 (36.5)
Non-Hispanic/non-Latino	719 (60.5)	372 (61.5)	371 (61.1)	739 (62.0)
Not reported	13 (1.1)	9 (1.5)	11 (1.8)	17 (1.4)
Age at vaccination, years				
Mean (SD)	44.3 (12.7)	44.5 (12.7)	45.1 (12.8)	45.3 (12.5)
Median (range)	46 (18–64)	46 (18–64)	46 (18–64)	46 (18–64)
Baseline SARS-CoV-2 status ^a^, *n* (%)				
Positive	1066 (89.7)	N/A	N/A	1066 (89.5)
Negative	115 (9.7)	N/A	N/A	116 (9.7)
Missing	8 (0.7)	N/A	N/A	9 (0.8)
Received previous influenza vaccination ^b^, *n* (%)	393 (33.1)	197 (32.6)	202 (33.3)	399 (33.5)
Number of previous influenza vaccinations ^b^, *n* (%)				
1	247 (20.8)	121 (20.0)	142 (23.4)	257 (21.6)
2	72 (6.1)	43 (7.1)	27 (4.4)	70 (5.9)
3	74 (6.2) ^c^	33 (5.5)	33 (5.4)	72 (6.0) ^d^
Received previous COVID-19 vaccination, *n* (%)	798 (67.1)	403 (66.6)	409 (67.4)	828 (69.5)
Number of previous COVID-19 vaccinations, *n* (%)				
1	150 (12.6)	84 (13.9)	79 (13.0)	162 (13.6)
2	255 (21.4)	128 (21.2)	123 (20.3)	278 (23.3)
3	206 (17.3)	94 (15.5)	103 (17.0)	190 (16.0)
4	112 (9.4)	49 (8.1)	65 (10.7)	122 (10.2)
5	45 (3.8)	32 (5.3)	27 (4.4)	54 (4.5)
6	27 (2.3)	15 (2.5)	10 (1.6)	19 (1.6)
7	2 (0.2)	0	1 (0.2)	2 (0.2)
8	0	1 (0.2)	1 (0.2)	1 (<0.1)
9	1 (<0.1)	0	0	0

BNT162b2, monovalent BNT162b2 (Omicron XBB.1.5) vaccine; modRNA, nucleoside-modified messenger RNA; N/A, not applicable; QIV, licensed quadrivalent influenza vaccine. ^a^ Baseline SARS-CoV-2 positivity required a positive N-binding assay result at baseline or a reported history of COVID-19. ^b^ In the previous 3 years only. ^c^ One participant received five previous influenza vaccinations. ^d^ One participant received four previous influenza vaccinations.

## Data Availability

Upon request, and subject to review, Pfizer will provide the data that support the findings of this study. Subject to certain criteria, conditions, and exceptions, Pfizer may also provide access to the related individual de-identified participant data. See https://www.pfizer.com/science/clinical-trials/trial-data-and-results for more information.

## Supplementary Materials

The following supporting information can be downloaded at: https://www.mdpi.com/article/10.3390/vaccines14080677/s1, Supplementary Text; Figure S1: Percentage of participants with HAI titers ≥ 1:40 before and 4 weeks after vaccination in Cohort 3; Table S1: Grading scale for local reactions, systemic events, and fever; Table S2: Median onset (time from vaccination, days) and duration (days) of local reactions and systemic events in Cohorts 2 and 3; Table S3: Serious AEs by preferred term reported in >1 participant in any group from vaccination through 6 months after vaccination in Cohorts 2 and 3; Table S4: AESIs reported from vaccination through 6 months after vaccination in Cohorts 2 and 3.

## Abbreviations

The following abbreviations are used in this manuscript:

AE	Adverse event
AESI	Adverse event of special interest
CBER	US Food and Drug Administration’s Center for Biologics Evaluation and Research
CHMP	European Medicines Agency’s Committee for Medicinal Products for Human Use
GMFR	Geometric mean fold rise
GMR	Geometric mean ratio
GMT	Geometric mean titer
HAI	Hemagglutination inhibition
LLOQ	Lower limit of quantification
modRNA	Nucleoside-modified messenger RNA
QIV	Quadrivalent influenza vaccine
SAE	Serious adverse event
