# Supplementary material for: Phase 3 Randomized Study Investigating the Safety, Tolerability, and Immunogenicity of a Combination Modified Messenger RNA Vaccine Against Influenza and COVID-19 in Healthy Adults
Source: Vaccines (Basel). 2026 Aug 5;14(8):677. doi: 10.3390/vaccines14080677 (PMC13517642; doi:10.3390/vaccines14080677)
Supplement: Supplementary file 1 [file vaccines-14-00677-s001.zip › Pineiro Puebla_Figure S1.pdf]

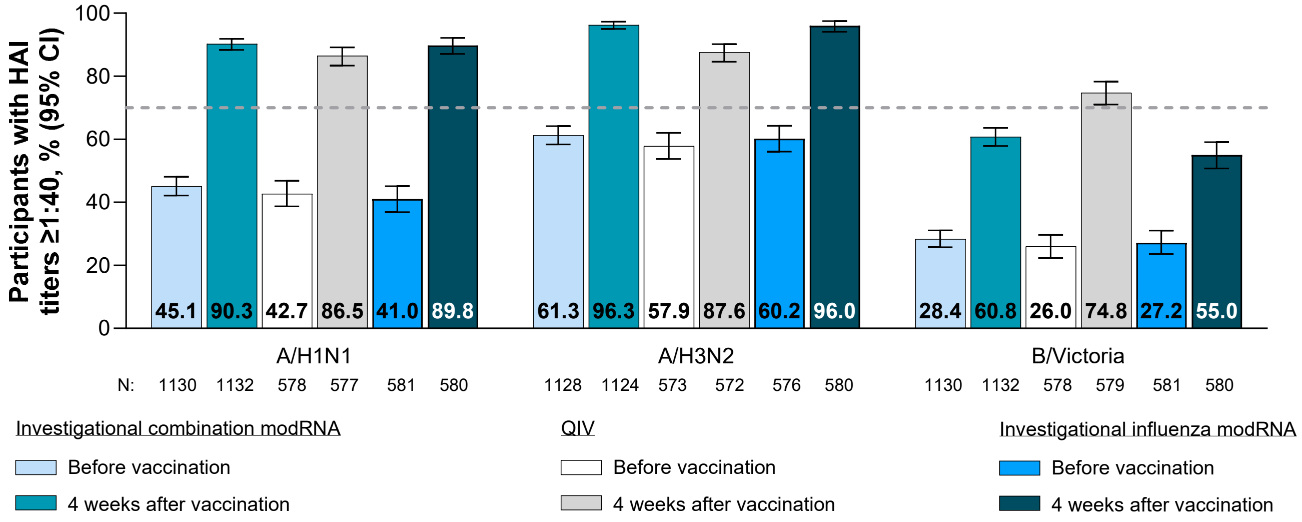

Figure S1. Percentage of participants with HAI titers ≥1:40 before and 4 weeks after vaccination in Cohort 3. The horizontal dashed line denotes the CBER/CHMP threshold of a lower bound of the 2-sided 95% CI of 70%. BNT162b2, monovalent BNT162b2 (Omicron XBB.1.5) vaccine; CBER, US Food and Drug Administration's Center for Biologics Evaluation and Research; CHMP, European Medicines Agency's Committee for Medicinal Products for Human Use; HAI, hemagglutination inhibition; investigational combination modRNA, investigational combination influenza plus COVID-19 modRNA vaccine; modRNA, nucleoside-modified messenger RNA; QIV, quadrivalent influenza vaccine.
