# Supplementary material for: Phase 3 Randomized Study Investigating the Safety, Tolerability, and Immunogenicity of a Combination Modified Messenger RNA Vaccine Against Influenza and COVID-19 in Healthy Adults
Source: Vaccines (Basel). 2026 Aug 5;14(8):677. doi: 10.3390/vaccines14080677 (PMC13517642; doi:10.3390/vaccines14080677)
Supplement: Supplementary file 1 [file vaccines-14-00677-s001.zip › Pineiro Puebla_Supplementary Text.pdf]

Supplementary Text

Eligibility criteria

Inclusion criteria

- Participants 18 through 64 years of age (or the minimum age of consent in accordance with local regulations) at baseline (ie, Visit 1).
- Participants who were willing and able to comply with all scheduled visits, investigational plan, laboratory tests, lifestyle considerations, and other study procedures.
- Healthy participants who were determined by medical history, physical examination (if clinically required), and clinical judgment of the investigator to be eligible for inclusion in the study. Healthy participants with preexisting stable disease, defined as disease not requiring significant change in therapy or hospitalization for worsening disease during the 6 weeks before enrollment, could be included.
- Capable of giving signed informed consent.

Exclusion criteria

- Any medical or psychiatric condition, including recent (within the past year) or active suicidal ideation/behavior or laboratory abnormality, that may increase the risk of study participation or, in the investigator's judgment, make the participant inappropriate for the study.
- History of severe adverse reaction associated with any vaccine and/or severe allergic reaction (eg, anaphylaxis) to any component of the study intervention(s).
- Immunocompromised individuals with known or suspected immunodeficiency, as determined by history and/or laboratory/physical examination.
- Bleeding diathesis or condition associated with prolonged bleeding that would, in the opinion of the investigator, contraindicate intramuscular injection.
- Females who were pregnant or breastfeeding.
- History of myocarditis or pericarditis.
- Receipt of systemic treatment with known immunosuppressant medications (including cytotoxic agents or systemic corticosteroids, eg, for cancer or an autoimmune disease), or radiotherapy, within 60 days before enrollment or planned receipt through conclusion of the study. Applied to long-term, high-dose systemic corticosteroids administered at a dose of ≥20 mg/day of prednisone or equivalent for ≥14 days. Low-dose systemic corticosteroids administered at a dose of <20 mg/day of prednisone or equivalent were permitted. Inhaled/nebulized, intra-articular, intrabursal, or topical (skin or eyes) corticosteroids were permitted.
- Receipt of blood/plasma products, immunoglobulin, or monoclonal antibodies used for the treatment or prevention of COVID-19 or those that were considered immunosuppressive, from 60 days before study intervention administration, or planned receipt throughout the study.
- Vaccination with any investigational or licensed influenza vaccine within 6 months (175 days) before study intervention administration, or ongoing receipt of chronic antiviral therapy with activity against influenza.

- Vaccination with any investigational or licensed COVID-19 vaccine within 6 months (175 days) before study intervention administration.
- Participation in other studies involving administration of a study intervention within 28 days prior to, and/or during, participation in this study.
- Investigator site staff directly involved in the conduct of the study and their family members, site staff otherwise supervised by the investigator, and sponsor and sponsor delegate employees directly involved in the conduct of the study and their family members.

Ethical study conduct

This study was completed in accordance with the protocol and consensus ethical principles derived from international guidelines, including the Declaration of Helsinki and Council for International Organizations of Medical Sciences International Ethical Guidelines, applicable International Council for Harmonisation of Technical Requirements for Pharmaceuticals for Human Use good clinical practice guidelines, applicable International Organization for Standardization 14155 guidelines, medical device guidelines, and other applicable laws and regulations, including privacy laws. The protocol, protocol amendments, and other relevant documents were reviewed and approved by the independent review board or ethics committee before enrollment. All participants provided written informed consent before enrollment. The study protocol and statistical analysis plan are available at ClinicalTrials.gov.
