# Supplementary material for: Phase 3 Randomized Study Investigating the Safety, Tolerability, and Immunogenicity of a Combination Modified Messenger RNA Vaccine Against Influenza and COVID-19 in Healthy Adults
Source: Vaccines (Basel). 2026 Aug 5;14(8):677. doi: 10.3390/vaccines14080677 (PMC13517642; doi:10.3390/vaccines14080677)
Supplement: Supplementary file 1 [file vaccines-14-00677-s001.zip › Pineiro Puebla_Table S1.pdf]

Table S1. Grading scale for local reactions, systemic events, and fever

Local reaction	Mild	Moderate	Severe	Grade 4
Injection-site pain	Does not interfere with activity	Interferes with activity	Prevents daily activity	ED visit or hospitalization for severe pain
Redness	>2.0–5.0 cm (5–10 measuring device units)	>5.0–10.0 cm (11–20 measuring device units)	>10 cm (≥21 measuring device units)	Necrosis or exfoliative dermatitis
Swelling	>2.0–5.0 cm (5–10 measuring device units)	>5.0–10.0 cm (11–20 measuring device units)	>10 cm (≥21 measuring device units)	Necrosis
**Systemic event**	**Mild**	**Moderate**	**Severe**	**Grade 4**
Vomiting	1–2 times in 24 hours	>2 times in 24 hours	Requires IV hydration	ED visit or hospitalization for hypotensive shock
Diarrhea	2–3 loose stools in 24 hours	4–5 loose stools in 24 hours	≥6 loose stools in 24 hours	ED visit or hospitalization for severe diarrhea
Headache	Does not interfere with activity	Some interference with activity	Prevents daily routine activity	ED visit or hospitalization for severe headache
Fatigue/tiredness	Does not interfere with activity	Some interference with activity	Prevents daily routine activity	ED visit or hospitalization for severe fatigue
Chills	Does not interfere with activity	Some interference with activity	Prevents daily routine activity	ED visit or hospitalization for severe chills
New or worsened muscle pain	Does not interfere with activity	Some interference with activity	Prevents daily routine activity	ED visit or hospitalization for severe new or worsened muscle pain
New or worsened joint pain	Does not interfere with activity	Some interference with activity	Prevents daily routine activity	ED visit or hospitalization for severe new or worsened joint pain
Fever	38.0°C–38.4°C (100.4°F–101.1°F)	>38.4°C–38.9°C (101.2°F–102.0°F)	>38.9°C–40.0°C (102.1°F–104.0°F)	>40.0°C (>104.0°F)

ED, emergency department; IV, intravenous.
