# Supplementary material for: Phase 3 Randomized Study Investigating the Safety, Tolerability, and Immunogenicity of a Combination Modified Messenger RNA Vaccine Against Influenza and COVID-19 in Healthy Adults
Source: Vaccines (Basel). 2026 Aug 5;14(8):677. doi: 10.3390/vaccines14080677 (PMC13517642; doi:10.3390/vaccines14080677)
Supplement: Supplementary file 1 [file vaccines-14-00677-s001.zip › Pineiro Puebla_Table S2.pdf]

Table S2. Median onset (time from vaccination, days) and duration (days) of local reactions and systemic events in Cohorts 2 and 3

	Cohort 2				Cohort 3							
	Investigational combination influenza plus COVID-19 modRNA vaccine (N=3111)		Concomitant QIV with BNT162b2 (N=1560)		Investigational combination influenza plus COVID-19 modRNA vaccine (N=1186)		QIV (N=604)		Investigational influenza modRNA vaccine (N=606)		BNT162b2 (N=1188)	
	Onset	Duration	Onset	Onset	Duration	Onset	Onset	Duration	Onset	Duration	Onset	Duration
Local reactions												
Redness	2.0	2.0	2.0	2.0	1.5	2.0	2.0	1.5	2.0	2.0	2.0	2.0
Swelling	2.0	2.0	2.0	2.0	2.0	2.0	2.0	2.0	2.0	2.0	2.0	2.0
Pain at the injection site	1.0	2.0	1.0	1.0	2.0	1.0	1.0	2.0	1.0	2.0	1.0	2.0
Systemic events												
Fever	2.0	1.0	2.0	2.0	1.0	3.0	2.0	1.0	3.0	1.0	2.0	1.0
Fatigue	2.0	2.0	2.0	2.0	2.0	2.0	2.0	2.0	2.0	2.0	2.0	2.0
Headache	2.0	2.0	2.0	2.0	1.0	2.0	2.0	1.0	2.0	1.0	2.0	1.0
Chills	2.0	1.0	2.0	2.0	1.0	2.0	2.0	1.0	2.0	1.0	2.0	1.0
Vomiting	2.0	1.0	3.0	3.0	1.0	3.0	3.0	1.0	3.0	1.0	2.0	1.0
Diarrhea	2.0	1.0	3.0	3.0	1.0	3.0	3.0	1.0	3.0	1.0	2.0	1.0
New/worsened muscle pain	2.0	1.0	2.0	2.0	1.0	2.0	2.0	1.0	2.0	1.0	2.0	1.0
New/worsened joint pain	2.0	1.0	2.0	2.0	1.0	3.0	2.0	1.0	3.0	1.0	2.0	1.0

BNT162b2, monovalent BNT162b2 (Omicron XBB.1.5) vaccine; modRNA, nucleoside-modified messenger RNA; QIV, quadrivalent influenza vaccine.
