# Supplementary material for: Phase 3 Randomized Study Investigating the Safety, Tolerability, and Immunogenicity of a Combination Modified Messenger RNA Vaccine Against Influenza and COVID-19 in Healthy Adults
Source: Vaccines (Basel). 2026 Aug 5;14(8):677. doi: 10.3390/vaccines14080677 (PMC13517642; doi:10.3390/vaccines14080677)
Supplement: Supplementary file 1 [file vaccines-14-00677-s001.zip › Pineiro Puebla_Table S3.pdf]

**Table S3. Serious AEs by preferred term reported in >1 participant in any group from vaccination through 6 months after vaccination in Cohorts 2 and 3**

| | Cohort 2 | | Cohort 3 | | | |
|---|---|---|---|---|---|---|
| | **Investigational combination influenza plus COVID-19 modRNA vaccine (N=3127)** *n* **(%)** | **Concomitant QIV with BNT162b2 (N=1563)** *n* **(%)** | **Investigational combination influenza plus COVID-19 modRNA vaccine (N=1189)** *n* **(%)** | **QIV (N=605)** *n* **(%)** | **Investigational influenza modRNA vaccine (N=607)** *n* **(%)** | **BNT162b2 (N=1191)** *n* **(%)** |
| Any serious AE | 23 (0.7) | 22 (1.4) | 10 (0.8) | 5 (0.8) | 7 (1.2) | 15 (1.3) |
| Osteomyelitis | 1 (<0.1) | 0 | 0 | 0 | 0 | 2 (0.2) |
| Pneumonia | 0 | 2 (0.1) | 0 | 0 | 0 | 0 |
| Pulmonary embolism | 1 (<0.1) | 2 (0.1) | 0 | 0 | 0 | 0 |
| Seizure | 0 | 2 (0.1) | 0 | 0 | 1 (0.2) | 0 |
| Subdural hematoma | 0 | 2 (0.1) | 0 | 0 | 0 | 0 |

AE, adverse event; BNT162b2, monovalent BNT162b2 (Omicron XBB.1.5) vaccine; modRNA, nucleoside-modified messenger RNA; QIV, quadrivalent influenza vaccine.
