# Supplementary material for: Phase 3 Randomized Study Investigating the Safety, Tolerability, and Immunogenicity of a Combination Modified Messenger RNA Vaccine Against Influenza and COVID-19 in Healthy Adults
Source: Vaccines (Basel). 2026 Aug 5;14(8):677. doi: 10.3390/vaccines14080677 (PMC13517642; doi:10.3390/vaccines14080677)
Supplement: Supplementary file 1 [file vaccines-14-00677-s001.zip › Pineiro Puebla_Table S4.pdf]

Table S4. AESIs reported from vaccination through 6 months after vaccination in Cohorts 2 and 3

	Cohort 2		Cohort 3			
	**Investigational combination influenza plus COVID-19 modRNA vaccine (N=3127)** ***n*** **(%)**	**Concomitant QIV with BNT162b2 (N=1563)** ***n*** **(%)**	**Investigational combination influenza plus COVID-19 modRNA vaccine (N=1189)** ***n*** **(%)**	**QIV (N=605)** ***n*** **(%)**	**Investigational influenza modRNA vaccine (N=607)** ***n*** **(%)**	**BNT162b2 (N=1191)** ***n*** **(%)**
Any	70 (2.2)	29 (1.9)	15 (1.3)	19 (3.1)	7 (1.2)	20 (1.7)
Infections and infestations	47 (1.5)	26 (1.7)	13 (1.1)	12 (2.0)	4 (0.7)	15 (1.3)
COVID-19	43 (1.4)	22 (1.4)	12 (1.0)	12 (2.0)	4 (0.7)	15 (1.3)
Influenza	4 (0.1)	5 (0.3)	1 (<0.1)	0	0	0
Reproductive system and breast disorders	23 (0.7)	3 (0.2)	2 (0.2)	7 (1.2)	3 (0.5)	5 (0.4)
Intermenstrual bleeding	2 (<0.1)	0	1 (<0.1)	1 (0.2)	0	0
Menstruation delayed	2 (<0.1)	0	1 (<0.1)	1 (0.2)	0	0
Heavy menstrual bleeding	10 (0.3)	1 (<0.1)	0	1 (0.2)	1 (0.2)	2 (0.2)
Hypomenorrhea	2 (<0.1)	0	0	0	1 (0.2)	1 (<0.1)
Menstruation irregular	3 (<0.1)	1 (<0.1)	0	2 (0.3)	0	1 (<0.1)
Oligomenorrhea	1 (<0.1)	0	0	1 (0.2)	0	0
Amenorrhea	3 (<0.1)	0	0	0	0	0
Menometrorrhagia	1 (<0.1)	0	0	1 (0.2)	1 (0.2)	1 (<0.1)
Menstrual clots	1 (<0.1)	0	0	0	0	0
Abnormal uterine bleeding	0	1 (<0.1)	0	0	0	0

BNT162b2, monovalent BNT162b2 (Omicron XBB.1.5) vaccine; modRNA, nucleoside-modified messenger RNA; QIV, quadrivalent influenza vaccine.
